# Ecological and Industrial Implications of Dynamic Seaweed-Associated Microbiota Interactions

**DOI:** 10.3390/md18120641

**Published:** 2020-12-14

**Authors:** Farid Menaa, P. A. U. I. Wijesinghe, Gobika Thiripuranathar, Bushra Uzair, Haroon Iqbal, Barkat Ali Khan, Bouzid Menaa

**Affiliations:** 1 Department of Nanomedicine, California Innovations Corporation, San Diego, CA 92037, USA; bmenaa@cic.com; 2 College of Chemical Sciences, Institute of Chemistry Ceylon, Rajagiriya 10107, Sri Lanka; pwijesinghe@ichemc.edu.lk (P.A.U.I.W.); tgobika@ichemc.edu.lk (G.T.); 3 Department of Biological Sciences, International Islamic University, Islamabad 44000, Pakistan; bushra.uzair@iiu.edu.pk; 4 Department of Pharmaceutics, College of Pharmaceutical Sciences, Soochow University, Suzhou 215123, China; hiqbal@suda.edu.cn; 5 Department of Pharmacy, Gomal University, Dera Ismail Khan 29050, Pakistan; barkat.khan@gu.edu.pk

**Keywords:** seaweeds, microbiome, holobiont, epibiosis, basibiont, environmental stress, quorum sensing, fouling, biofilm disruption, secondary metabolites

## Abstract

Seaweeds are broadly distributed and represent an important source of secondary metabolites (e.g., halogenated compounds, polyphenols) eliciting various pharmacological activities and playing a relevant ecological role in the anti-epibiosis. Importantly, host (as known as basibiont such as algae)–microbe (as known as epibiont such as bacteria) interaction (as known as halobiont) is a driving force for coevolution in the marine environment. Nevertheless, halobionts may be fundamental (harmless) or detrimental (harmful) to the functioning of the host. In addition to biotic factors, abiotic factors (e.g., pH, salinity, temperature, nutrients) regulate halobionts. Spatiotemporal and functional exploration of such dynamic interactions appear crucial. Indeed, environmental stress in a constantly changing ocean may disturb complex mutualistic relations, through mechanisms involving host chemical defense strategies (e.g., secretion of secondary metabolites and antifouling chemicals by quorum sensing). It is worth mentioning that many of bioactive compounds, such as terpenoids, previously attributed to macroalgae are in fact produced or metabolized by their associated microorganisms (e.g., bacteria, fungi, viruses, parasites). Eventually, recent metagenomics analyses suggest that microbes may have acquired seaweed associated genes because of increased seaweed in diets. This article retrospectively reviews pertinent studies on the spatiotemporal and functional seaweed-associated microbiota interactions which can lead to the production of bioactive compounds with high antifouling, theranostic, and biotechnological potential.

## 1. Introduction 

The ocean is the mother of life and it harbors a vast variety of marine organisms which are diverse in their physiology and adaptations [1]. Algae (macro- and micro-) constitute anything from 30,000 to more than 1 million different species, with a great diversity of forms and sizes [2]. Despite uncertainties regarding what organisms should be included as algae and what a species is in the context of the various algal phyla and classes, an attempt is being made to arrive at a more accurate estimate of algal species [2]. Algae are best defined as “oxygenic photosynthesizers other than embryophyte land plants” [3]. Indeed, they are evolutionary quite diverse and do not represent a single taxonomic entity as compared to vascular plants which can be assigned into a single Phylum called Tracheophyta [3]. Indeed, depending on the systematics and molecular phylogeny, they roughly belong to four kingdoms: Kingdom Plantae (e.g., chlorophytes and rhodophytes—green and red algae, respectively), the Kingdom Protozoa (ex-Protista) (e.g., Euglenozoa, ex-Euglenophytes), the Kingdom Chromista (e.g., phaeophytes aka brown algae—including dinoflagellates and diatoms), and the Kingdom Eubacteria (cyanophytes, as known as blue-green algae) [3,4]. Actually, according to the International Code of Botanical Nomenclature (ICBN) (https://www.iapt-taxon.org/nomen/main.php), the algae have been classified into 11 divisions, i.e., Cyanophycophyta (blue-green algae), Chlorophycophyta (green algae), Charophyta (stoneworts), Euglenophycophyta (Euglenoids), Xanthophycophyta (yellow-green algae), Chrysophycophyta (golden algae), Bacillariophycophyta (diatoms), Phaeophycophyta (brown algae) currently included in Ochrophyta (brown and golden-brown algae), Pyrrophycophyta, Cryptophycophyta, Rhodophycophyta (red algae) [2]. It is worth noting that the euglenoids (class of Euglenophyceae) secondarily acquired a green algal chloroplast by symbiogenesis, and recently became well classified as algae and not anymore as protozoan flagellate Euglenoidina as it was the case in the 20th century [3]. Nevertheless, concerns arise because of the decline of taxonomists worldwide that has a tremendous repercussion on the improvement and completion of necessary systematic studies [2]. 

Seaweeds (macroalgae), mainly located on the coastline, represent a considerable part of the ocean biomass (estimate of 25,000–30,000 species) [2]. They have always been used as food and feed by the coastal populations due to their high nutritional composition [5,6]. In ancient times, traditional methodologies have been used to cultivate seaweeds; however, in the past 50 years, the development of cultivation methodologies (e.g., sea-based cultures, cultures in sea water ponds) tremendously increased their production [7]. 

Based on the chemical composition (e.g., pigments), seaweeds are classified into green algae, brown algae, and red algae. Thereby, brown seaweeds contains pigments of fucoxanthin and chlorophyll a and c; red seaweeds contain pigments of phycoerythrin, allophycocyanin, xanthophylls, and chlorophyll a, while green seaweeds contain pigments of xanthophylls and chlorophyll a and b [5,8]. 

Seaweeds also contain an excellent source of bioactive compounds including complex polysaccharides (structural and storage polysaccharides), sulfated polysaccharides [9,10], as well as polyphenols, minerals, carotenoids, amino acids (glycine, arginine, alanine, and glutamic acid) [11], proteins/peptides (phycobiliproteins, glycoprotein, phycolectins, and mycosporine) [11], water-soluble fibers [12], fat-soluble vitamins (i.e., A, D, E, and K), macro-minerals (Na, K, Ca, and Mg) in addition to trace elements (e.g., Fe, Zn, Mn, and Cu) [5,13]. However, they contain a small amount (1–5%) of lipids, mainly polyunsaturated fatty acids (PUFAs); brown and red algae containing more eicosapentaenoic acid (EPA) and arachidonic acid (AA) than green algae [13]. It is important to mention that the chemical composition of seaweeds varies depending on the environmental factors such as salinity, geographical habitat, seasonal variation, and ambient conditions such as water temperature, nutrient concentration, light intensity, and ocean acidification [14,15,16,17]. As a consequence, some of the bioactive compounds produced by algae have attracted much interest in the food [5], pharmaceutical [18,19], biomedical [10], biotechnological [20], agricultural [1], aquacultural [21], and energy [22,23] industries. For instance, red algae (Rhodophyta) are rich at least in one type of the water-soluble sulfated polysaccharide carrageenan, which is known to exert antitumor and antiviral activities besides their wide uses in the food industry as thickeners, stabilizers, and emulsifiers [11,24]. Additionally, another sulfated polysaccharide extracted from brown seaweeds (Phylum Ochrophyta, Class Phaeophyceae), namely fucoidan, was found to elicit antiviral, anticancer, and anticoagulant activities [25]. Polysaccharides and peptides extracted from different seaweeds exerted prebiotic effects, regulating the intestinal epithelial cell, macrophage, and lymphocyte proliferation/differentiation processes as well as the immune response [25]. Seaweeds are a valuable source of vitamins (e.g., B_12_, C, E) and pigments (carotenoids) which exert antioxidant activities (vitamins A, C, and β-carotene), decrease blood pressure (vitamin C), reduce the effects of aging and anemia (vitamin B_12_), prevent from cardiovascular diseases (β-carotene), support the vision (vitamin A) [1,3,5,12,13]. In the last three decades, interest has grown in seaweed-derived phytochemicals as nutraceuticals, functional foods, and therapeutics to protect against superoxidation and inflammatory diseases (e.g., diabetes, cancers, cardiovascular diseases) [3,11,12,25].

Seaweeds are widely distributed along the coastal areas worldwide and form rich marine meadows and forests [26]. The temporal variation and distributional pattern of algal assemblage can be used as bioindicators that herald the reflection of self-adaption for the ecosystem to the possible changes in coastal environments [27,28]. Changes in hydrodynamic forces have a major effect on the algal diversity, abundance, distribution of macroalgae, algae–microbiota interactions, and epiphytic fauna (e.g., grazers) [29]. Seaweeds are structuring species in coastal zones, changing light, stabilizing the sediment, and modifying the hydrodynamic ecosystem [30,31]. Moreover, the submerged macroalgal beds enhance the biodiversity, supporting complex food webs by providing habitats, food, reproductive refugia to diverse organisms (e.g., mammals, seabirds, fish, invertebrates) [32]. Several studies addressed the effects of spatial and temporal variations that induce physical disturbance in macroalgae species [33,34,35].

Interestingly, the halobiont concept, which came into the limelight 10 years ago, suggests a mapping of all interactions and activities within and between a host and all its associated organisms/epiphytic species (e.g., bacteria, fungi, archaea, viruses, diatoms, and other unicellular organisms) which together form a discrete functional ecological unit [36,37]. Marine ecosystems are based on multiple interactions among organisms which may be competitive, mutualistic, parasitic, or symbiotic [38,39]. Macroalgal surfaces are coated by an organic layer, due to adsorption of organic and inorganic molecules, allowing them to harbor a rich diversity of associated microorganisms such as bacteria, fungi, diatoms, other unicellular organisms, spores, and larvae of marine invertebrates [40]. Due to the importance and pervasiveness of marine algae, there has been a strong scientific interest in elucidating halobionts in a dynamic spatial-temporal and functional context, not only for an ecological purpose but also for an industrial interest (e.g., targeted and controlled production of bioactive compounds). Many attempts are made to elucidate the precise roles of intercellular chemical signaling pathways that regulate the dynamic interactions between algae and associated epiphytic or endophytic microbiota. Since the last decade, emerging methods to better understand algal–microbial ecology using high-throughput screening (HTS) and robust metagenomic analyses are enabling (i) faster identification of algal species, microcosms [41,42,43], (ii) the build of well-supported phylogenies improving our understanding of how horizontal transfer has influenced the evolution of the algal genomes [44]. 

Furthermore, quorum sensing (QS) is a system of chemical signaling which gains increasing attention from marine ecologists [45]. QS occurs within microbial populations in a density-dependent manner, causes downstream changes in the gene regulation, and modulates many biological functions like virulence factor expression, biofilm formation, bioluminescence, sporulation, and bacterial conjugation [24,46,47]. According to the literature, epiphytic bacterial communities are pivotal in the normal morphological development of the algal host, and their antifouling capacities by QS would protect chemically undefended macroalgae (those unable to deliver defense strategies themselves) from detrimental and secondary colonization by macroscopic epibiota [48,49,50]. 

This review focused on the major interactions between most common microorganisms (e.g., bacteria) associated with marine macroalgae. We highlight the spatiotemporal distribution and function of these halobionts, with a special emphasis on how environmental stress factors (biotic and abiotic) influence the maintenance, stability, and establishment of halobionts. We also address the potential impact of algal–microbiota interactions in the production of secondary metabolites which are valuable both from an ecological and industrial point of view. 

## 2. Seaweed-Associated Microorganisms Interactions: An Overview 

The Earth’s microbial diversity is mainly concentrated in the ocean which undeniably represents a great reservoir of bioactive substances [51]. Marine microorganisms (e.g., bacteria, fungi, other unicellular organisms, diatoms, spores, and larvae of marine invertebrates) play crucial roles in every marine ecological process, hence the growing interest in studying their populations and functions [52]. 

Intriguingly, the surface of seaweeds provides a suitable substratum for the settlement of microorganisms particularly because it secretes various organic substances that function as nutrients for the formation of microbial biofilms [53]. The colonization of microorganisms on the seaweed surface is extraordinarily complex and dynamic, because the abundant and diverse epiphytic microbiota (e.g., Proteobacteria, Firmicutes) play a crucial role in morphogenesis and growth of seaweeds in direct and/or indirect ways [38,39,54].

The host-associated microbiota is found to be tissue-specific [52,55]. Other biotic factors involved in the algal halobionts include interactions among bacterial taxa (i.e., both internal and from the surrounding water), consequences of biological interactions with organisms from other trophic levels (i.e., grazing, cross-feeding) and also the loss of certain functions by bacteria [56].

Marine macroalgae are typically the home to aerobic and photoautotrophic epibionts (i.e., epiphytic organisms, those living at the surface of seaweeds) or endobionts (i.e., endophytic organisms, those living within the seaweeds). Thereby, epiphytic bacteria are found at densities varying from 10^2^ to 10^7^ cells·cm^−2^ depending on the macroalgal species, season, and thallus section [38,57]. In recent years, researchers have paid more attention on characterizing microbiomes (epibionts and endobionts) of various algal species in order to explain the host–microbe interactions and also to understand the function of microbial communities [58]. Nevertheless, it is worth noting that most of the experimental studies have focused on epibacterial communities and little attention has been focused on the diversity of fungi, endophytic and other epiphytic organisms [1,2,3]. This is most likely due to the higher representation of the bacterial community on algae, which halobiont is one of the easiest to study. For instance, nitrogen-fixing Cyanobacteria were recently observed to be among the dominant active members of the microbial community associated with the red seaweed *Laurencia dendroidea* (J.Agardh) [59]. 

According to several studies, the type of algae–bacteria interactions could be classified either as (i) nutrient exchange, (ii) signal transduction, or (iii) gene transfer [59]. Thereby, the participation of epiphytic bacterial communities include [47,52,55,60] (i) production of bioactive compounds, which protect the host (algae) from harmful entities present in the pelagic realm by determining the presence of other bacterial strains; (ii) provision of other effective molecules (e.g., vitamins among other nutrients), which are responsible for morphology, development, and growth of seaweeds; (iii) consumption of organic matter and nitrogen (NO_3_^−^) source; (iv) defense through QS (by Gram negative bacteria)/antifouling chemicals. 

Further, algae–bacteria interactions cover the whole range of symbiotic relationships, which are mainly identified as mutualism, commensalism, and parasitism [61,62]. It is important to mention that environmental factors (e.g., Azote (N):Phosphorus (P) ratio, light intensity, temperature, pH, salinity) may shift an interaction from one type to another [62]. 

Mutualism is a biologic interaction in which two or more partners of different species benefit each other [62,63]. A typical example of mutualism is that a bacterial species supplies vitamin B_12_ (cobalamin) to an algal partner in exchange for fixed carbon (C) [64]. Thereby, the freshwater green microalga *Lobomonas rostrata* is auxotrophs for the vitamin B_12_ which is supplied for its growth promotion by the Gram-negative bacterium *Mesorhizobium loti* (formerly known as *Rhizobium loti*) [65,66,67].

Commensalism can be defined as an intraspecific relationship in which the commensal obtains great benefits (e.g., food, shelter, or locomotion) from the host without causing adverse effects [62,68]. For instance, the microalga *Chlamydomonas reinhardtii* (Phylum Chlorophyta) uses vitamin B_12_ delivered by heterotrophic bacteria, although the bacteria do not make use of the organic carbon released by the alga [67].

Parasitism is an interaction in which one species (i.e., commonly, the parasites) benefits at the expense of the other (i.e., usually, the alive algae) and exerts negative effects on it [62]. It has been estimated that some algae (about 10% of known red algae) are parasitic [69]. Interestingly, many bacteria are known to “negatively” affect algae growth rates, which in fact is encouraging for scientists who strive to control algae blooms [39,58,61,70]. This effect occurs either by competition for nutrients, altruism, or through algal cytolysis which is mediated by the action of bacterial glucosidases, chitinases, cellulases, and other enzymes (pectinases), allowing the bacteria to use the intracellular algal compounds as nutrients [62,71]. Such parasites have also wide-ranging applications in industrial biotechnology (e.g., pharmaceutical, food, alcoholic beverages, paper, and/or textile industries) [72,73].

In terms of algal–bacteria interactions, it is worthwhile to mention that the presence of carbon-rich constituents of macroalgal cell walls (e.g., agar, carrageenan, alginate, fucoidan, laminarin, cellulose, and pectin), are likely to be important for bacterial colonization [3,74,75]. Indeed, parasitic bacteria are likely to supply cell-wall degrading enzymes as a mechanism to mobilize polymers for nutritional purposes [60]. It has been proposed that macroalgal-polysaccharide-degrading bacteria increase in numbers on weakened or dead macroalgae, thereby contributing to recycling of the algal biomass [76]. Several algal polysaccharide-degrading bacteria displaying hydrolytic activities, such as Flavobacteria and γ-Proteobacteria (Gram-negative bacteria), have recently been isolated from the microflora of the brown seaweed *Ascophyllum nodosum* (Linnaeus) [59]. The functional screening of plurigenomic libraries from these bacteria resulted in a range of novel hydrolytic enzymes [60]. Bacteria with polymer-degrading traits may thus represent opportunistic pathogens or saprophytes, rather than commensal or mutualistic macroalgal symbionts [77]. Further, some epiphyte microorganisms harbor a partial cellulosome, such as the marine psychrophilic bacterium *Pseudoalteromonas tunicate* (D2), which lacks the enzymes required to hydrolyze macroalgal cell wall polymers, although they still contain the structures involved in polymer binding [60]. *P. tunicata* also maintains the capability to utilize monomers derived from the degradation of typical macroalgal polymers, such as cellulose and xylan (a group of hemicelluloses), thereby benefiting from other microorganisms once its host is compromised [38]. It is also interesting to note that *P. tunicate* produced biologically active compounds such as the antibacterial protein alpP, known to be effective against a great number of Gram-negative and Gram-positive bacteria from a range of environments, thereby conferring a competitive advantage to *P. tunicate* during the biofilm growth on algal surfaces [78]. 

Although studies on seaweeds–microbionts interactions are attracting growing interest there is still a paucity of reports about seaweeds and other microorganisms, such as fungi [4,5,79]. Briefly, it was reported that the algal–fungal interactions commence with spore attachment and hyphal invasion, resulting in colonization of either parasitic, mutualistic, endosymbiotic, or saprophytic fungi [6]. These algal-inhabiting fungi are called algicolous, and those associated with the thallus surface are termed as fungal epiphytes or epibiotic fungi. Due to their ecological significance, algicolous fungi gain a particular interest in recent research. However, details on the distribution and occurrence of algicolous fungi are incomplete [7]. Thus, it will be an asset to explore the structure and dynamics of these fungal assemblages to understand the ecology of fungal–algal interactions among the numerous other seaweed-associated microorganisms that exist in the marine ecosystem. 

In summary, this variety of exchanges between biotic communities in aquatic ecosystems, has an impact that can range from beneficial to detrimental effects on the algal growth and the environment (e.g., cycling of NO_3_^−^. The control of halobionts may serve as an incredible useful tool to control (i) the production of a given algal species, thereby avoiding harmful algal blooms, (ii) feed animals (e.g., pelagic fishes), and/or (iii) harvest algal biomass at a low cost for industrial considerations [62,80]. 

## 3. The Multifaceted Roles of Seaweed-Associated Bacteria: Friends or Foes?

### 3.1. Bacteria Supply Key Nutrients and Are Required for Normal Morphological Development of Seaweeds

Epiphytic heterotrophic bacteria supply key substances, such as carbon dioxide (CO_2_) and fixed NO_3_^−^, required for macroalgal photoautotrophy, growth, and survival [59,81].

Epiphytic bacteria may also assist in or complement the macroalgal host’s primary production since autotrophic cyanobacteria are often abundant on benthic macroalgal species [58]. 

In addition, epiphytic bacteria have a positive impact on the morphological development of several macroalgal species. Indeed, certain green macroalgae do not develop normal morphology in the absence of native bacterial communities. Specifically, the axenically grown green seaweed *Ulva australis* (Areschoug), formally known as *Ulva pertusa* (Kjellman) and commonly named sea lettuce, developed an abnormal ‘pincushion’-like morphology, which could be restored to the typical foliose thallus upon reinoculation with bacterial strains isolated from the alga [38,39]. Similar effects have been reported for other species of green seaweeds, including, *Ulva linza* (Linnaeus), formally known as *Enteromorpha linza*, *Ulva compressa* (Linnaeus), formally known as *Enteromorpha compressa*, *Ulva fasciata* (S.F.Gray), and *Gayralia oxysperma* (Kützing) K.L. Vinogradova ex Scagel and al., formally known as *Monostroma oxyspermum* (Kützing) [7,39,82]. 

In addition to epiphytic bacteria, endophytic bacteria such as endophytic actinobacteria play helpful roles for seaweeds. Actinobacteria are widely found in sediments or in association with macroalgae among other marine organisms, such as fish, sponges, corals, and tunicates [73,83]. Such endophytic microorganisms live in the inner tissues of plants and algae without causing negative damages to the host [58]. During this symbiotic association, endophytes produce secondary metabolites that improve the fitness of the host and its resistance against environmental stressors, obtaining in return nutrients and shelter from their host [20]. 

### 3.2. Microorganism-Mediated Biofouling: Ecological Significance for Seaweeds and Their Antibiofouling 

Biofouling is the undesirable accumulation of microorganisms, plants, algae, and animals on wetted surfaces. The bacterial-mediated biofouling represents the “Achilles heel” due to bacteria’s ability to multiply over time, forming a very dangerous pervasive biofilm, including on seaweeds’ surface [84,85]. These bacterial biofilms display a complex 3D structure made of a consortium of bacterial species which are encased in an extracellular polymeric substance matrix comprising biomacromolecules (e.g., polysaccharides) and humic substances [86,87]. 

Studies of the epiphytic microbial communities present on macroalgae (10^2^–10^7^ cells·cm^−2^) [38,45,57,88] have accentuated the spatial distribution of bacteria, with specific parts of the thallus hosting specific bacterial populations. In some cases, the bacterial populations even change with the season or the age of the host [88,89]. Further, these bacterial biofilms facilitate the attachment and growth of a range of other fouling organisms, namely diatoms, invertebrate larvae, and algal spores [88,90]. 

Therefore, bacterial biofouling is recently gaining much interest due to its severe economic (e.g., annual loss estimated to USD 6.5 million), and environmental adverse (e.g., release of CO_2_ and sulphur dioxide (SO_2_)) effects) [85]. Indeed, bacterial biofouling can physically damage the host organism (e.g., through production of toxins, digestive enzymes, and waste products by the microbial communities) and cause biological competition leading to environmental modifications [91]. Bacteria use specific appendages to bind to the host (e.g., macroalgal) surface [20,45]. Attached epiphytes bacterial populations must then compete with each other for nutrients and space through antifouling mechanisms (e.g., production of antagonist chemical metabolites/antibiotics) [92,93]. Thereby, the transcriptome analysis of microbiome associated with the red alga *L. dendroidea* revealed an overexpression of extracellular polysaccharides [59,93]. Thus, the balance between the bacterial biofouling and antifouling, through the production of bioactive substances from microbial (e.g., bacterial) strains that produce bioactive substances within the host (e.g., macroalgal) surface, largely contribute to the ecosystem’s dynamism [60]. Interestingly, biofouling can lead to the production of biologically active compounds/chemicals by either the epiphytic microorganisms and/or the host organism when it can control the biofouling. Thereby, infochemicals (e.g., phenolics) produced on the algal surface can act as a chemical defense against pathogens and other microorganisms that compete for nutrients (e.g., mucilage) [37]. For instance, actinobacteria isolated from macroalgae can produce bioactive compounds, including antibiotics, antitumor, and anti-inflammatory compounds. In this regard, our team has made a recent breakthrough discovery by isolating an original antibiotic, effective against MRSA clinical strains, originated from a new epibiotic actinobacterium that we named *Kocuria marina* CMG S2 which was using the brown seaweed *Pelvetia canaliculata* (Linnaeus) as a basibiontic organism (i.e., providing substrate for other organisms) [73]. Additionally, the marine bacterium *P. tunicata*, frequently found associated with the green seaweed *U. australis*, produce a diverse range of biologically active compounds against common fouling organisms [88,94]. Indeed, as earlier evoked, *P.* *tunicata* produce the antibacterial protein alpP, which in the context of biofilm growth provided a competitive advantage to *P.* *tunicata* when tested in laboratory [78,88]. Since *U. australis* has no known physical or chemical defense systems against fouling organisms, it has been consequently suggested that the host may manipulate the bacterial community on its surface, which in turn protects the host by interfering with the development of a mature biofouling community [88]. Thereby, within the bacterial community related to *U. australis*, abundant multidrug-efflux pumps and nonribosomal peptide synthetases were found to be frequently involved in the production of bioactive substances, further supporting the role of chemically mediated antagonism and counteractive defense processes in such marine environments [55,95]. 

Besides, some other algae, such as tropical marine brown macroalgae members of the Fucales (e.g., *Turbinaria ornata* (Turner) and *Sargassum pacificum* (Bory), formally known as *Sargassum angarevense* (Grunow)), were able to directly produce bioactive compounds (e.g., fatty acids, lipopeptides, glucids, pigments, amides, alkaloids, lactones, steroids, terpenoids, pyrroles, halogenated metabolites) which are generally assumed to function as chemical defenses against herbivores/grazers, bacteria, other undesirable epiphytic associations, and even as inducible screens against UV radiation [49,96,97,98]. Thus, these secondary antibiofouling metabolites represent a robust selective factor for epiphytic bacterial colonizers, influencing the bacterial biofilm formation and its community composition [38,45,99,100,101]. 

Major microorganismal antifouling (i.e., antimicrobial colonization, inhibition of biofilm formation) compounds (e.g., brominated, polyphenolic) produced by macroalgae are summarized in Table 1. 

### 3.3. Disturbance of the Macroalgal Halobiont by Bacterial Pathogens: The Crucial Role of Quorum Sensing

Macroalgae are the major habitat formers and they contribute to the primary production in temperate marine ecosystems [116]. Unfortunately, there is some evidence to suggest that microbial disease is a possible factor contributing to the (i) decline of macroalgal populations, (ii) environmental changes, such as increase of seawater temperatures which impact halobionts, (iii) reduction of innate defense strategies in macroalgal hosts, and (iv) susceptibility to colonization and infection of macroalgae by pathogens [52,113]. 

Suitable models are being developed to address the specific virulence mechanisms employed by seaweed pathogens [13]. The bacterial-induced bleaching of the red alga *Delisea pulchra* (Greville) caused by the bacterium *Nautella italica* R11 (formerly *Ruegeria* sp. R11), a member of the Roseobacter clade, is one of the best-studied models for disease in macroalgae [31,117]. The infection process of *N. italica* R11 in the chemically defended marine macroalga *D. pulchra* was shown to be temperature dependent [118].

There seems to be a link between QS and diseases through the regulation of certain phenotypes. Therefore, it is thought that the induction of virulence factors are liable for pathogen–host association [81]. Several reports indicated that numerous aquatic organisms like microalgae, macroalgae, invertebrates, or maybe other bacteria have the potential to disrupt QS [119]. Importantly, the disruption of bacterial communication by QS is considered as a novel and environmentally friendly approach to avoid harmful consequences for the algal health and fecundity. The mechanism of action varies from degradation of signals through enzymatic or chemical inactivation to antagonistic and agonistic activities [120]. Thereby, QS systems, such as the classical LuxR-type QS, mediate the host colonization and coordinate the expression of virulence genes in pathogenic bacteria [50]. For instance, in *N. italica* R11, it was demonstrated that the acylated-homoserine lactone (AHL) signal molecule, produced in a LuxI-dependent manner, binds a unique response regulator (LuxR)-type gene, *varR*, which modulates the expression of biofilm-associated proteins that control its colonization, persistence, and virulence on the surface of a macroalgal host [50]. Importantly, such a *varR* gene with homology to LuxR-type transcriptional regulators was present in another bleaching-associated disease pathogen, namely *Phaeobacter* sp. LSS9 [45]. 

Furthermore, it has also been established that macroalgae-associated bacterial isolates produce signal molecules, like N-acyl homoserine lactone (AHLs) [48], thereby facilitating the settlement of zoospores in seaweeds such as in the green macroalgae *Ulva* spp. [45]. Deeper investigations about zoospore settlement revealed that the orientation of zoospore does not change during the settlement but the mechanism underlining this phenomenon has not been clearly reported yet [45]. However, it has been assumed that AHLs influence Ca^2+^ in fluxin zoospore which preferentially induces the settlement through chemokinesis [121]. To date, there is limited knowledge about the significant role of the cross-kingdom QS signaling between associated bacterial communities and carpospore liberation from red macroalgae [45,48].

## 4. Spatial-Temporal Exploration and Functional Distribution of the Macroalgal Halobiont

### 4.1. Climate Changes and Other Environmental Stresses-Induced Diversity in Algal and Related Halobionts 

Apart from the biotic variables (e.g., halobionts, presence of grazer species [39,122,123,124]), natural abiotic variables are liable for the decrease or increase in the growth of algal species and their wide marine diversity [125]. Indeed, the seaweed species are spatiotemporally distributed along the coastal areas worldwide (e.g., North Western Europe) [26], and there is increasing evidence that their interactions with the microbiota and epifauna, their biogenic habitat (e.g., shipwrecks, other types of substrata), their growth, development (acquired blade size morphology, floating structures/pneumatocysts), and abundance (e.g., high density sometimes leading to blooms) depend on abiotic factors such as changes in salinity, pH, temperature, sun radiation, and nutrients [122,123,124,126,127,128,129]. Thus, algae can be then used as bioindicators that herald doable changes in coastal environments. In this regard, the fast spreading/invading green seaweed *Caulerpa taxifolia* (M.Vahl) represents a great example of a broad ecological plasticity which makes it a potential threat for the Mediterranean benthic communities, being responsible, at least in part, for the decay and regression of the seagrass *Posidonia oceanica* (Linnaeus) [130,131]. 

The temporal variation and distributional pattern of seaweeds are the reflection of self-adaption for the ecosystem (e.g., reproduction, growth, longevity, distribution, abundance, and interactions of algae with epiphytic microorganisms, epiphytic fauna, seagrass) to the changing conditions [29,31,35]. Additionally, seaweed species are periodically subjected to continual storms, temperature variation, abrasion, and other stresses which accelerate community succession [122,132]. Hydrodynamic forces have a major effect on the distribution patterns of rocky shore organisms, and its magnitude determines both the abundance and the diversity of macroalgae, not to include epiphytic faunas [14,30]. For instance, benthic macroalgae, such as the large brown algae *Macrocystis* spp., may become detached from primary substratum as a consequence of different biotic and abiotic factors but continue to live for extended periods of time after detachment, maintaining positive buoyancy (for weeks to months) and even reproductive structures (i.e., sporophylls) [122,123]. The dominant floating seaweeds in the world’s oceans are brown algae (class of Phaeophyceae), belonging to the genera *Macrocystis*, *Fucus*, *Sargassum*, *Ascophyllum*, *Durvillaea*, *Carpophyllum*, *Phyllospora*, and *Cystophora* [123]. Besides, the submerged macroalgal beds increase the biodiversity through providing habitat for marine organisms [30,31]. The dynamics of floating seaweeds in a particular area are determined by different factors, such as size and location of sources, temporal supply dynamics, floating potential at the sea surface, winds, currents, wave surges, and other hydrographic features such as frontal systems that drive dispersal, accumulation, and sink processes [26,122,126,127].

Biological, chemical, and physical properties on the macroalgal surface are likely to play a key role in structuring the associated microbial community and its metabolic activity in both qualitative and quantitative manner [20]. For instance, a first community-scale investigation demonstrated a quantitative divergence in phylogenetically unrelated bacterial communities on the surfaces of eight closely related sympatric kelp species from four sites in British Columbia. The kelp forest-associated bacterial diversity would be correlated with the life-history strategy of the host, and not with its phylogeny [129]. Structure, composition, and mutualistic relations of the seaweed-associated microbiota are known to change with the ecological pressures/environmental stresses, host conditions, space, and time [129,133].

**Figure 1:**
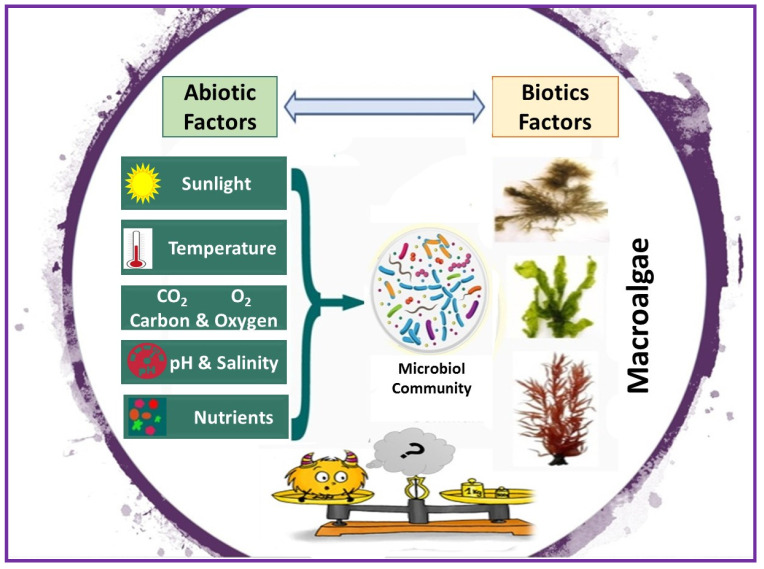
Influence of environmental stressors on macroalgal halobionts.

Seasonal variations (climate change) are likely related to the combined effect of biotic and abiotic factors [134]. Indeed, climate changes generally impact macroalgae due to high concentrations of atmospheric CO_2_, which in turn induce a rise in temperature and eventually contribute to a decline in pH in seawater [135]. Thermal stress contributes to slower growth rates and at sublethal temperature can cause deterioration of the tissue [136]. The different environmental abiotic stresses (e.g., significant changes in light, temperature, salinity, nutrient starvation) would cause change in the microbiota by disrupting their symbiotic relationships with algae (Figure 1). Therefore, a disequilibrium in an aquatic environment can be roughly summarized by complex unbalanced interactions between abiotic and biotic factors.

### 4.2. Influence of Major Abiotic Factors in the Regulation of Macroalgal Halobionts

**Figure 2:**
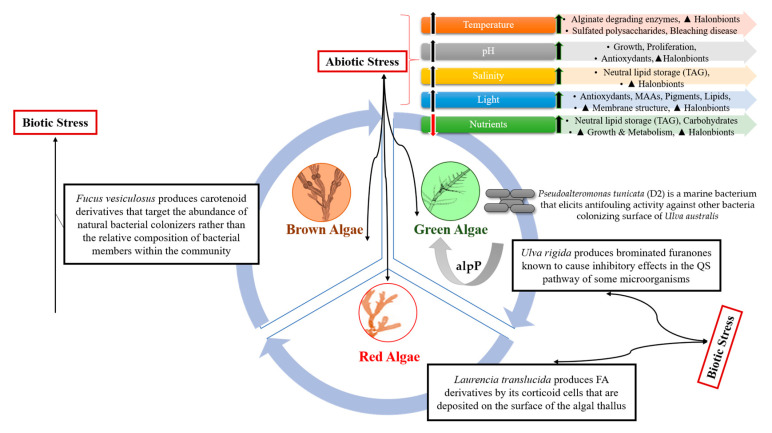
Holistic picture of biotic and abiotic stressors versus macroalgae’s defense strategies. TAG: triacylglycerol; MAAs: mycosporine-like amino acids; QS: quorum-sensing; FA: fatty acid.

The complex interactions underlying macroalgal halobiont(s) responses to climate change and other environmental stressors may be driven by shifts in the microbiota (Figure 2). However, it is worth mentioning that such interactions across aquatic ecosystems remain largely unresolved, illustrated by the lack of studies published in that field. The fact that the macroalgae-associated microbiota interactions may be altered by abiotic factor-mediated drastic changes are being hypothesized based on similar studies with microalgae. Interestingly, one of the key factors driving these responses is the infochemical-mediated communication in the halobiont which is crucial in driving halobiont survival, adaptation, and/or halobiont breakdown [37]. 

#### 4.2.1. Effect of the Temperature on the Assemblage and Maintenance of Algal Halobionts 

Temperature plays an important role in photoinhibition, consequently being one of the most important environmental factors influencing the algal size, growth rate, biochemical composition, availability of nutrients as well as the algal–microbial interactions [137].

Experiments with the marine diatom *Phaeodactylum tricornutum* (Bohlin) exposed to a range of temperatures (25 to 10 °C) showed that lowering culture temperature could significantly raise yields of long chain PUFAs, mainly EPA, docosahexaenoic acid (DHA), and alpha-linoleic acid (ALA) [138].

The thermal plasticity of macroalgae is based on their capacity to adapt to temperature changes; nevertheless, this acclimation under thermal stress mainly depends on the algal species and the persistency of the heat stress. Thereby, variable thermal responses were observed among macroalgal traits and biogeographic regions [139,140,141]. Thereby, when seawater temperatures are elevated, it is assumed that the macroalga’s chemical defense is weakened, leading more frequently during summer to bleaching disease in the temperate red seaweed *D. pulchra* [52].

Besides, it was recently reported that NO_3_^−^ sufficiency enhanced thermal tolerance in the habitat-forming kelp *Macrocystis pyrifera* (Linnaeus), ameliorating the negative impacts on physiological performance (i.e., growth and photosynthesis) [139]. 

Interestingly, an independent study experimentally demonstrated that the water and kelp microbiome responded differently to the independent or combined effects of increased temperatures and partial pressure of CO_2_ (*p*CO_2_) used as common global climate stressors in the ocean [142]. Specifically, the kelp microbiome, most influenced elevated temperature in combination (or not) with elevated *p*CO_2_, exhibited a reduction in Alteromondales, the dominant kelp-associated order. Additionally, the kelp growth was negatively associated with elevated temperature, and the kelp microbiome showed increases in Flavobacteriales, alginate degrading enzymes and sulfated polysaccharides. In contrast, kelp growth was positively associated with the combination of high temperature and high *p*CO_2_ ‘future conditions’, with great increases in commensal microbes (i.e., Planctomycetales and Rhodobacteriales) that responded to an increased mucus production. Altogether, this was an example that a macroalgae can stabilize the microbiome under changing *p*CO_2_ conditions but can lose control at high temperature. 

#### 4.2.2. Effect of the pH on the Assemblage and Maintenance of Algal Halobionts 

pH is another global stressor which affects the algal growth and development. Although the impact of CO_2_-driven ocean acidification (OA) on algae and macroalgal halobionts has been relatively well studied, the populational responses to this extremely sensitive factor may vary due to the transgenerational physiological plasticity.

pH determines the solubility and the availability of both CO_2_ and essential nutrients [143], and in case of drastic changes, it can significantly influence the algal metabolic processes [144]. Indeed, dissolved inorganic carbon (DIC) levels can stimulate the algal growth and specific production of compounds (e.g., anti-herbivore, photo-protective molecules) [145,146]. 

Although it was reported that a group of coralline red macroalgae, namely rhodoliths, (Corallinales, Rhodophyta), are particularly susceptible to OA due to reduced calcification rates as carbonate ions decrease [147], a recent study showed that, under high *p*CO_2_ conditions, live rhodoliths exhibited positive physiological responses (e.g., increased photosynthetic activity, no calcium carbonate biomass loss over time) and the tightly regulated microbial–host interaction was evidenced as stable and healthy, which is considered as important for host resilience to environmental stress [148]. This most recent study is in agreement with the fact that since the essential substratum for carbon fixation is CO_2_, many macroalgae species may get advantage from OA such as increased growth, proliferation, production of antioxidants, and/or photosynthetic rates [149]. Thereby, other findings strengthen the evidence that calcified brown macroalgae, such as *Padina pavonica* (Linnaeus) (Phaeophyceae, Dictyotales) and noncalcified brown macroalgae *Cystoseira compressa* (Esper) (Phaeophyceae, Fucales) can benefit physiologically from increases in DIC as the oceans acidify, but that benefits (i.e., proliferation, higher contents in carbon and antioxidants (e.g., chlorophyll a, phenols, fucoxanthin)) as well as the extent of the algal response, depend upon nutrient and light availability [150]. Taken together, macroalgal responses to OA may well depend upon their nutrient metabolism, which can vary widely between species [147].

#### 4.2.3. Effect of the Salinity on the Assemblage and Maintenance of Algal Halobionts 

Salinity is another essential factor that modifies the biochemical composition of algal cells and the algal–microbiota interactions. This abiotic factor can influence the biotic abundance, growth, and community composition. Salinity can be then an overall driver of ecosystem function and is considered as one of the most influential environmental determinants, not only for distribution of benthic and pelagic organisms but also for the microbial community composition [151,152,153]. For instance, salinity can change community structure and ecological function in Archaea and affect bacterial abundance, growth, and activity [154,155]. 

High salinity is a challenge for marine organisms to proliferate and even survive [156]. Contemporary studies of differential expression have started to evaluate in microalgae the pathways that are regulated differently under optimal and high salt stress conditions [157]. Thereby, marine microalgae can adapt different mechanisms to survive high salt stress, mainly by changing their metabolism [158]. To cope with salinity issue, most algal cells commonly produce and accumulate lipids as a possible algal strategy to strengthen their membranes. Thereby, the marine green microalga *Dunaliella tertiolecta* (Butcher), including high triacylglycerol (TAG) content in a highly concentrated saline environment [159].

However, the influence of salinity as a driver of epibacterial community composition (until species level) has been very rarely investigated for seaweeds, especially under long time scales. 

A recent study, that examined, through the use of metagenomic sequencing, the influence of various salinities (low, medium, and high) at different times (i.e., 3 and 5 months) on a sampling of epibacterial community of an invasive red seaweed *Agarophyton vermiculophyllum* (Ohmi) Gurgel, J.N. Norris et Fredericq, formerly *Gracilaria vermiculophylla* (Ohmi), revealed that the epibacterial richness varied both irrespectively of the salinity levels and time points [160]. Thus, it has been concluded by the authors that both salinity and time can be major driving forces in structuring epibacterial communities of seaweeds with respect to richness and diversity.

#### 4.2.4. Effect of the Light on the Assemblage and Maintenance of Algal Halobionts 

During the process of photoautotrophic/holophytic development organisms, algae can synthesize their own food (organic compounds) from inorganic substances using light as an energy source. Since the light intensity and spectral composition of the incident light vary worldwide, with heavy local and cyclic dependence, the photosynthesis capacity and subsequent growth rate of algae species is expected to vary. 

In fact, the photoadaptation/photoacclimation of algal cells allows them to change their properties according to the light availability [161]. Thereby, algal cells resolve the light restriction by undertaking changes in chloroplast membranes while an increase in light intensity over saturation limits induces photoinhibition and changes in the algae’s cell structure. This is the case of the green microalga *Dunaliella tertiolecta* (Butcher) which showed a decrease in protein content and an amplified lipid production with increased light intensities up to its saturation [162]. Many seaweeds have even developed buoyant gas-filled/swim bladders (as known as pneumatocysts), floating structures that hold fronds upright in the water column and enhance their access to light for photosynthesis [163]. Further, marine macroalgae, especially the red ones (e.g., *Bostrychia scorpioides* (Hudson) Montagne, *Porphyra dioica* (J.Brodie and L.M.Irvine), *A. vermiculophyllum* (Ohmi) Gurgel, J.N. Norris et Fredericq, and *Vertebrata lanosa* (Linnaeus) T.A. Christensen), developed defense mechanisms including the synthesis of photoprotective molecules (e.g., pigments such as chlorophyll a, phycobiliproteins and carotenoids; mycosporine-like amino acids (MAAs) such as shinorine, palythine, usurijene, and palythene) against light and particularly harmful UV radiation [164]. 

Importantly, UV radiation not only affects the physiology of seaweeds but also their interactions with microorganisms. Thereby, a study that aimed to determine changes in abundance and composition of epibiotic bacteria on the agarophyte *Gelidium lingulatum* (Kützing) exposed to UV for 5 days. UV exposure changed the composition of microbial communities on the thalli and led to a reduction in community diversity and evenness (e.g., density of *Alteromonas* sp. increased but abundances of *Methylophaga* sp. and *Colwellia* sp. decreased) [165]. Another study reported the effect of light on the heterotrophic activity in biofilms of the brown macroalgae *Sargassum furcatum* Kützing during its growth cycle [166]. The biofilm composition was mainly represented by bacteria, but diatoms, cyanobacteria, and other organisms were also common. It was concluded that primary producers in the biofilm are more important to increase bacterial activity than the macroalgae itself because of coherence of the peaks of heterotrophic and autotrophic (e.g., mostly diatom genera-mediated) activities in biofilms during the artificial extinction of natural light and the effects of autotrophic inhibitors on heterotrophic activity.

#### 4.2.5. Effect of the Nutrients on the Assemblage and Maintenance of Algal Halobionts

To achieve maximum growth and ensure photosynthesis of lipids, proteins, and carbohydrates, the aquatic organisms including algae must be supplied or acquire nutrients in a stoichiometrically balanced manner [167].

It has been reported that the physicochemical conditions of the substratum (e.g., rock, sand with pebbles, cobbles and boulders, sediment, mud, dead matte, biogenic hard substrate) are directly associated to the enrichment of nutrient availability [131,168,169].

Nutrients required by algae, which influence their the biochemical composition, can be classified as (i) mineral macronutrients (e.g., NO_3_^−^, phosphorus, sulfur, potassium, and magnesium); (ii) mineral micronutrients (e.g., iron and manganese needed in small amounts (30–2.5 ppm), and trace elements (4.5–2.5 ppm) such as cobalt, zinc, boron, copper, and molybdenum); (iii) nonmineral nutrients (e.g., carbon, hydrogen, and oxygen), (iv) polymers [125]. 

Further, it is worth noting that algae (e.g., kelp) forest ecosystems are biodiversity hotspots, providing habitat for dense assemblages of marine organisms and nutrients for marine and terrestrial food webs. Thereby, surfaces of algae support diverse microbial communities that facilitate the transfer of carbon from algal primary production to higher trophic levels [129]. 

Among the mineral macronutrients, NO_3_^−^ and phosphate are two important macronutrients for growth and metabolism of algal cells, being fundamental elements for the structure and function of proteins and nucleic acids; phosphorus is besides essential for the formation of ATP, the energy carrier in cells, as well as a key component of phospholipids [170]. The starvation of NO_3_^−^ in many natural environments, makes the algae shift their lipid metabolism from membrane lipid synthesis to neutral lipid storage (such TAG), subsequently increasing total lipid content in algae [171,172]. Besides, phosphorus starvation decreases chlorophyll and protein content, thus increases the relative carbohydrate content of algal cells [173]. The availability of these nutrients is thought to be an essential factor regulating the growth of toxic algal blooms [174].

Among the mineral micronutrients, trace metals such as Iron (Fe), manganese (Mn), cobalt (Co), zinc (Zn), copper (Cu), and nickel (Ni) are considered as vital for the algae because they are involved in a range of metabolic functions [175]. Interestingly, the algae cell surfaces contain a variety of functional groups (e.g., sulfhydryl, carboxylic, and phosphatic groups) that adsorb with strong affinity metallic protons [176]. Importantly, restriction of these trace metals (especially Cu, Ni, and Fe) can negatively affect the algal growth and the production of antioxidants (e.g., carotenoids) [177]. Conversely, exceeded concentrations (above the threshold) of these metals can also inhibit growth, damage photosynthesis, and increase the lipid content. It is worth mentioning that Fe is particularly prominent in biochemical (redox) catalysis in photosynthesis of all the trace metals [178]. Thus, limitations in Fe (found under the forms of Fe(II) and Fe(III)) significantly depress the photosynthetic electron transfer, resulting in a reduction in NADPH formation [179]. Limiting Fe also decreased cellular chlorophyll concentration in algae [180]. 

Among the nonmineral nutrients, carbon (C) and oxygen (O) are two critical elements for algae’s elemental bioprocesses. C is necessary for photosynthesis, respiration, reproduction, growth, and metabolism of algae. C, supplied in the form of carbon dioxide (CO_2_), bicarbonate (HCO_3_), or carbonate (H_2_CO_3_), depending on the pH of the seawater (normally averaged to 8.2), can be used for autotrophic growth while for heterotrophic growth is rather used in the form of acetate or glucose [125]. A significant effect of the water-soluble CO_2_ coming from the atmosphere has been noticed on the composition of PUFAs, pigments, proteins, and carbohydrates. Thereby, lower CO_2_ concentrations in *Emiliania huxleyi* (Lohmann) W.W. Hay and H.P. Mohler (Haptophyta, Coccolithophyceae), the most common calcified microalgae on Earth, led to an increase in 22:6 (n−3) PUFA, whereas 14:0 fatty acids (FAs) were found to be predominant at higher CO_2_ concentrations [181]. Besides, when the cyanobacterium *Arthrospira* (formerly, *Spirulina*) *platensis* (Gomont) registered high concentrations of CO_2_, it was noticed a reduction in the maximum biomass yield through decreased production of pigments and proteins although the carbohydrate content was increased [182].

Seaweeds produce O_2_ as a byproduct of photosynthesis [183]. Interestingly, the surface of deep-sea macroalgae could represent a selective habitat for ammonium-oxidizing bacteria [39] found on it in relative abundance (1% of total bacteria) [39,184]. However, when macroalgae must defend themselves against attack of bacterial epiphytes, they can rapidly release large amounts of harmful reactive oxygen species (ROS) (e.g., superoxide ions (O^−^) and hydrogen peroxide (H_2_O_2_)) [61,185,186]. In turn, to minimize damages, resident bacteria can express enzymatic-based defenses (e.g., peroxidase, catalase, and other oxidases) that degrade ROS [185,187]. Although the importance of this defense system has not been directly established yet, the microbial metagenome of *U. australis* and the transcriptome of the microbial community associated with *L. dendroidea*, demonstrated that several macroalgal-associated bacteria may contain an abundance of genes associated with the oxidative stress response [187,188].

Eventually, the macroalgae’s cell wall constituents contain polymers (e.g., polysaccharides such as carrageenan, cellulose, agar, and alginate) which are essential nutrients for the bacterial colonization, hence participate in the modulation of microbiota interactions [189]. Thereby, Goecke et al. provided an insight to different enzymatic activities occurring in marine bacteria which destroy cell walls of macroalgae, which was then detrimental to the host and fully beneficial to the microbiota [190]. Additionally, it is interesting to remind that bacteria associated to macroalgae long term may lack the capability to initial polymer degradation. Thereby, as previously evoked, the dark-green-pigmented marine epiphytic bacterium *P. tunicata* lack the enzyme required to hydrolyze polymers of macroalgae cell wall, although the structures required for polymer binding remained present [60,191]. Thus, polymer-degrading bacteria can reflect opportunistic pathogens instead of commensal or mutual macroalgal symbionts. Further, macroalgae defend themselves by producing secondary metabolites such as halogenated furanones which interfere with cell–cell signaling systems in several bacteria [192]. 

## 5. Industrial Benefits of Macroalgal-Halobionts Produced Secondary Metabolites

The best understanding of the aquatic ecosystems, in particular the interactions between macroalgae and bacteria (which appears to be the most representative community), will allow us to identify, isolate, and high-scale produce original second metabolites (e.g., antibiotics, antioxidants, anticancer compounds, polysaccharides, pigments, fatty acids, peptides) potentially interesting for a number of industrial applications (e.g., biofuels, fertilizers, drugs, cosmetics, nutraceuticals, wastewater treatment, food) [19,73,193,194,195]. 

A large sustainable source of chemical macroalgal-derived compounds with bioactivities was most recently reviewed [196]. For instance, the diverse and complex bacterial communities associated with *A. nodosum* represent a potential source of novel hydrolytic enzymes with biotechnological (e.g., cosmeceutical, functional food, nutraceutical, and biopharmaceutical) applications [83,197].

Additionally, polyphenolic metabolites extracted from the brown kelp *Ecklonia maxima* (Osbeck), known as “Phlorotannins”, contain high potential health benefits through various bioactivities. These polyphenols, also encountered in other brown algae belonging to the family of Fucaceae, are particularly well known for their unique anti-HIV activity by inhibiting ribonuclease enzyme activity, antimatrix metallopeptidases (MMPs), anti-inflammatory, and antioxidant properties [198,199].

Additionally, new bromophenols extracted from the marine red alga *Symphyocladia latiuscula* (Harvey) Yamada exhibited potent competitive tyrosinase inhibitory activity against l-tyrosine substrates. Interestingly, these isolated tyrosinase inhibitors exhibited an antibrowning property and thus, could be use as whitening agents [200].

**Figure 3:**
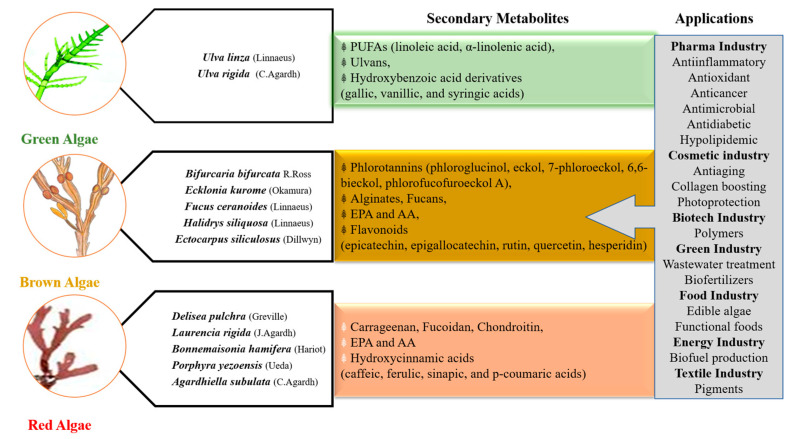
Examples of macroalgal-derived secondary metabolites and main applications in different industries. PUFAs: polyunsaturated acids; EPA: eicosapentaenoic acid; AA: arachidonic acid.

Eventually, many major secondary metabolites produced by the three phyla of macroalgae (i.e., Rhodophyta, Ochrophyta, and Chlorophyta), and not necessarily secreted or involved in antifouling activities, present a relative benefit for medicinal and industrial usage (Figure 3).

## 6. Conclusions and Perspectives

In this article, we reviewed the diverse and complex macroalgal halobionts with a special emphasis on the tightly regulated macroalgae-associated bacteria interactions, which is the most studied up to date certainly because macroalgal-epiphytic bacteria are the most representative microbial community. The concept of ‘halobiont’ emerged a decade ago and it is largely assumed that the diverse microbial associations to algae can be either beneficial or detrimental. We also highlighted the antifouling systems necessary to maintain a relative equilibrium in the macroalgal-halobionts, which mainly depend on both biotic and abiotic factors. It is worth noting that there is still a paucity of studies related to halobionts-related metagenomics, spatiotemporal distribution, and habitat types of macroalgal halobionts as well as on the influence of abiotic factors on the same (including the impact of water and sediment pollution caused by human actions). In the context of global climate change that includes rising temperature and increased *p*CO_2_ concentrations in the ocean, studies to evaluate the potential impacts on marine organisms (macroalgae) and their interactions are continuously needed with the expectation that the portfolio of natural substances, valuable both from an ecological and industrial point of view, will be enhanced. Indeed, although increasing evidence showed that environmental stress may disturb the complex mutualistic macroalgae-associated microbiota relations, it is difficult to predict how environmental stress will affect the macroalgal halobionts in a dynamic changing ocean.

## Figures and Tables

**Table 1:** Antifouling compounds produced by some macroalgae from a different phylum.

Macroalgae	Class (Secondary Metabolite(s))	References
**Green algae**		
*Ulva rigida* (C.Agardh)	Brominated furanones (3-bromo-5-(diphenylene)-2(5*H*)-furanone)	[102]
*Ulva* sp.	Tetraterpenoids (β-carotene)	[103]
**Brown algae**		
*Dictyota menstrualis* (Hoyt)	Diterpenes (dictyol D, pachydictyol A)	[104]
*Sargassum* spp.	Polyphenols (phlorotannins)	[105]
*Bifurcaria bifurcata* (R.Ross)	Acyclic linear diterpenoids (eleganediol, eleganolone, geranylgeraniols)	[106]
*Fucus vesiculosus* (Linnaeus)	Fucoxanthin (carotenoid/epoxycarotenol)	[107]
*Lobophora variegata* (J.V.Lamouroux)	Cyclic lactone (lobophorolide)	[108,109]
*Canistrocarpus cervicornis* (Kützing)	Diterpenes (dolastane, *seco*-dolastane)	[110]
**Red algae**		
*Laurencia* sp. (J.V.Lamouroux)	Omaezallene derivatives, sesquiterpenes (omaezol, hachijojimallene A, elatol)	[111]
*Laurencia translucida* (Fujii and Cordeiro-Marino)	Fatty acids (docosane; hexadecane)	[112]
*Asparagopsis taxiformis* (Delile) Trevisan	Sulfonate/dodecanoic acid (FA)-derived (2-dodecanoyloxyethanesulfonate)	[46]
*Bonnemaisonia hamifera* (Hariot)	Poly-brominated ketone (1,1,3,3-tetrabromo-2-heptanone)	[98]
*Delisea pulchra* (Greville)	Brominated furanones (*N*-acyl-homoserine lactones-like compounds)	[113]
*Callophycus serratus* (Harvey ex Kützing)	Bromophycoic acids (Bromophycolides and callophycoic acids)	[114]
*Mastocarpus stellatus* (Stackhouse)	Glucids (Floridoside)	[115]

FA: fatty acid.

## Abbreviations

AA	Arachidonic acid
ALA	Alpha-linoleic acid
C	Carbon
CO_2_	Carbon dioxide
DIC	Dissolved inorganic carbon
DHA	Docosahexaenoic acid
EPA	Eicosapentaenoic acid
FAs	Fatty acids
QS	Quorum sensing
MAAs	Mycosporine-like amino acids
N	Azote
NO_3_	Nitrogen
O	Oxygen
OA	Ocean acidification
P	Phosphate
pCO_2_	Partial pressure of CO_2_
PUFAs	Polyunsaturated fatty acids
ROS	Reactive oxygen species
